# Oral hyperpigmentation as an initial clinical aspect of hand foot syndrome

**DOI:** 10.1590/0103-6440202204711

**Published:** 2022-08-26

**Authors:** Éder Gerardo dos Santos-Leite, Lorena Vieira Sacramento, Alessandra Monteiro Santana, Juliana Borges de Lima Dantas, Manoela Carrera, Gabriela Botelho Martins

**Affiliations:** 1 Piracicaba Dental School, University of Campinas, Piracicaba, Brazil.; 2 Dental School Federal University of Bahia. Salvador, Bahia, Brazil.; 3 Program in Interactive Processes of Organs and Systems at the Institute of Health Sciences of the Federal University of Bahia. Salvador, Bahia, Brazil.; 4 Adventist College of Bahia. Bahiana School of Medicine and Public Health, Salvador, Bahia, Brazil.; 5 Department of Life Sciences at the State University of Bahia, Salvador, Bahia, Brazil.; 6 Faculty of Dentistry, Federal University of Bahia, Salvador, Brazil; 7 Multidisciplinary Institute of Rehabilitation and Health. Program in Interactive Processes of Organs an Systems at the Institute of Health Sciences at the Federal University of Bahia, Salvador, Bahia, Brazil.

**Keywords:** oral mucosa, hyperpigmentation, capecitabine, hand-foot syndrome, antineoplastic agent

## Abstract

Hand-foot syndrome (HFS) is a common adverse effect of anticancer therapy. It is known to cause dermatological symptoms including acral erythema and dysesthesia of the palms and soles of the feet, swelling, pain, itching, and scaling. Some drugs, like capecitabine, are known to trigger this condition. However, pigmentation of the oral mucosa is a rare adverse effect. This study aims to report a case of oral mucosa hyperpigmentation caused by capecitabine therapy before the clinical diagnosis of HFS. A 58-year-old female, diagnosed with invasive breast duct carcinoma, had the central nervous system, liver, skin, and lung metastasis, using capecitabine every day for 14 cycles. Oral examination revealed multifocal black macules on the hard palate, bilateral buccal mucosa, gingival mucosa, and dorsum of the tongue. The clinical hypothesis was oral mucosa hyperpigmentation by capecitabine use and only periodic follow-up was necessary. Hyperpigmentation of oral mucosa by capecitabine is a rare consequence of neoplastic therapy and your association with HFS is unclear, and poorly reported. The report of these events is important to alert oncology health teams about the individual tolerance to capecitabine therapy.

## Resumo

A síndrome mão-pé (SMP) é um efeito adverso comum da terapia anticâncer. Sabe-se que causa sintomas dermatológicos, incluindo eritema acral e disestesia das palmas das mãos e solas dos pés, inchaço, dor, coceira e descamação. Alguns medicamentos como a capecitabina são conhecidos por desencadear essa condição. No entanto, a pigmentação da mucosa oral é um efeito adverso raro. Este trabalho tem como objetivo relatar um caso de hiperpigmentação da mucosa oral causada pela terapia com capecitabina antes do diagnóstico clínico de SMP. Mulher de 58 anos, com diagnóstico de carcinoma invasivo de ducto mamário, apresentou metástase no sistema nervoso central, fígado, pele e pulmão, em uso de capecitabina todos os dias por 14 ciclos. O exame oral revelou máculas negras multifocais no palato duro, mucosa bucal bilateral, mucosa gengival e dorso de língua. A hipótese clínica foi de hiperpigmentação da mucosa oral pelo uso de capecitabina e apenas o acompanhamento periódico foi necessário. A hiperpigmentação da mucosa oral pela capecitabina é uma consequência rara da terapia neoplásica e sua associação com SMP não é clara e pouco relatada. O relato desses eventos é importante para alertar as equipes de saúde oncológica sobre a tolerância individual à terapia com capecitabina.

## Introduction

Capecitabine is an oral chemotherapeutic agent frequently used in the treatment of patients with metastatic breast cancer that can also be indicated as a first-line treatment for other malignant neoplasms, with emphasis on metastatic colorectal cancer and advanced gastric cancer ^1^ ^,^ ^2^ ^,^ ^3^. This drug is a prodrug rapidly absorbed by the liver and therefore being converted to 5-flourouracil (5-FU) through thymidine phosphorylase enzyme in tumor tissue, where it is abundantly expressed ^4^. One of the most common side effects associated with the use of chemotherapeutic agents such as capecitabine, is the presence of Hand-Foot Syndrome (HFS), characterized, among other signs, by skin hyperpigmentation, hyperkeratosis and palmar-plantar desquamation ^4^ ^,^ ^5^. However, some researchers report hyperpigmentation in other anatomical sites such as the ear, malar region ^6^, trunk, neck ^7^ and oral cavity ^3^ ^,^ ^4^ ^,^ ^8^ .

Hyperpigmentation of the oral mucosa related to the administration of chemotherapy drugs, in particular, capecitabine, is still underreported in the literature, and its pathogenesis is ill-defined. In addition, previous studies have questioned whether hyperpigmentation of the oral mucosa is yet another manifestation of HFS or whether it behaves differently ^3^ ^,^ ^4^. Furthermore, pigmentation of the oral mucosa due to capecitabine is not listed as a side effect on the drug leaflet (XELODA®: Capecitabine. F. Hoffmann-La Roche Ltd.). Due to the difficulty in diagnosis and the scarcity of reports in the literature, the aim of the present study was to report a case of hyperpigmentation of the oral mucosa in a cancer patient diagnosed with invasive metastatic breast ductal adenocarcinoma, submitted to oral capecitabine (XELODA®) as an antineoplastic therapy.

## Case report

**Figure 1:**
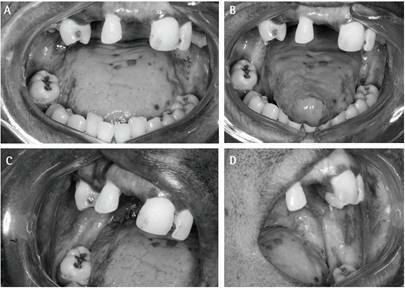
Multiple blackened - dark macules of different shapes in the oral cavity. (A) Blackened - dark macules in the back regions of the tongue and mucosa of the maxillary anterior alveolar ridge; (B) Blackened - dark macules on the back of the tongue; (C) and (D) blackened - dark macules in the region of the right and left buccal mucosa, close to the retromandibular region.

**Figure 2:**
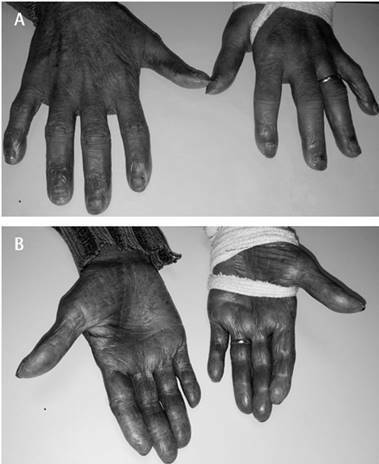
(A and B) Back and palm aspects showing diffuse blackish color.

A 58-year-old black female, with a history of alcoholism and smoking for approximately 30 years, and abstinence for 18 years, attended the dental office at a referral hospital, referred by the radiotherapist in May 2018, complaining of dryness of the oral mucosa. Through intraoral physical examination, there were multiple blackened - dark macules in the hard palate, bilateral buccal mucosa, upper and lower gingival mucosa and dorsum of the tongue (Figure 1), however, with no reported discomfort. In addition, through extraoral physical examination, the patient also presented diffuse black palmar pigmentation (Figure 2). At this time a clinical hypothesis of mucosal hyperpigmentation due to antineoplastic drug was raised, but required further investigation. Therefore, as an initial conduct, artificial saliva, and lip balm were prescribed for complaints of dry mouth.

The patient´s medical history consists of a diagnosis of invasive ductal adenocarcinoma on the right breast (T_2_N_3_M_x_) in 2015. She was initially submitted to unilateral mastectomy and axillary lymphadenectomy, followed by adjuvant chemotherapy using the ACT protocol (Anthracycline, Cyclophosphamide and Taxane) until November 2015, followed by radiotherapy (RT), 5040cGy (28 fractions of 180cGy in right plastron and 4500 cGy in 25 fractions of 180cGy in the right clavicular fossa), from March 2016 to April 2016. The ACT protocol was suspended due to disease progression and therapy with oral chemotherapeutic drugs, tamoxifen and anastrozole was instituted, associated with RT in the plastron region. Two years after the initial diagnosis, she presented with disease recurrence in the left orbit and in the plastron, being subjected to an orbitectomy. In 2018, the patient was diagnosed with metastasis in the central nervous system and liver, when she underwent a new RT in the head and neck region. In the same year, two new metastasis were found in skin and lungs, when oral capecitabine (XELODA®) was prescribed. The recommended dose was 500mg / twice a day for 14 days, followed by a rest week. After 14 cycles, she showed low tolerance to therapy with asthenia and diarrhea. For that reason, the drug was suspended to adjust the dose, and then, the patient was diagnosed with grade 1 HFS by the oncologist. At this time, she attended the Dental office, when hyperpigmentation of the oral mucosa due to chemotherapeutic agent diagnosis was closed, a condition that was present before the diagnosis of HFS by the medical team. From the diagnosis, the conduct established by the dentistry team was the periodic clinical monitoring of the oral spots. The patient was followed for a period of 22 months and died due to malignancy in July 2020.

## Discussion

Hand-Foot Syndrome, also known as palmar-plantar erythrodysesthesia, is considered to be one of the adverse effects of capecitabine, with a poorly understood pathophysiology and limited information regarding its treatment and prevention ^9^ ^,^ ^10^ ^,^ ^11^. Acral erythema and dysesthesia of the palms of the hands and soles of the feet are characteristic signs of the syndrome, starting with a tingling sensation and progressing to burning sensations, ulceration, and severe pain ^4^ ^,^ ^12^ ^,^ ^13^. Dermatological symptoms include painful erythema and edema, itching, dysesthesia, which may be followed by dry or moist desquamation of the palms and the soles. In severe cases, there is cracking, flaking, peeling of skin, blisters, ulcers, and severe pain ^14^.

The National Cancer Institute (NCI) classifies the disease in three stages according to the level of severity as described in Table 1 ^15^. The patient in the present report was staged as grade 1, considered the mildest. HFS still behaves as a reversible clinical condition with compromised quality of life, regularly requiring modification of therapeutic management or discontinuation of therapy until syndrome remission ^3^ ^,^ ^11^ ^,^ ^12^ ^,^ ^13^. Discontinuation of capecitabine for a few days or weeks usually leads to the disappearance of HFS’ signs and symptoms, however, this depends on the degree and severity of the lesions ^16^. This interruption may last for weeks or may extend until the patient returns to stage 0 or 1 HFS. As the patient in this case had HSF grade 1, the drug was discontinued for a few days and then was readjusted and reinserted upon stabilization of the syndrome, which could explain why no significant regression of pigmented lesions were observed in the mouth. The possible of persistency and progression of the lesions, was described by Lassere and Hoff ^15^.

**Table 1:** HFS grading according to National Cancer Institute (NCI).

NCI grade	NCI definition
1	Skin changes or dermatitis without pain (e.g., erythema, peeling).
2	Skin changes with pain, not interfering with function.
3	Skin changes with pain interfering with function.

Based in criterial defined by Nagore et al., 2000 ^15^.

The pathophysiology of HFS remains a matter of controversy. Several studies have theorized mechanisms that seek to explain the disease. Sanghi et al. ^17^ hypothesize that the products of 5-FU metabolism, rather than the drug itself, may be responsible for HFS. Thymidine phosphorylase, expressed in large amounts in tumor tissues, is also found in high concentrations in the palms of the hands and soles of the feet which, when found in these regions, could explain the clinical presentation of HFS. Other theories such as the excretion of capecitabine by eccrine sweat glands, local trauma of diverse origin causing rupture of small vessels leading to drug extravasation and consequent soft tissue damage, and mitochondrial dysfunction that causes cell apoptosis and keratinocytes death. This fact suggests a decrease of the corneal layer in patients with HFS in prolonged use of capecitabine ^17^ ^,^ ^18^ ^,^ ^19^. In view of the few reports in the literature, the theory that best explains the pathophysiology of HSF cannot be prioritized.

There are currently few cases of capecitabine-induced hyperpigmentation published in the literature ^3^ ^,^ ^4^ ^,^ ^6^ ^,^ ^7^ ^,^ ^8^. In the present report, the patient is a black-skinned woman who presents hyperpigmentation of the oral mucosa, hands, and feet, associated with continuous and prolonged use of capecitabine. In his study, Tognetti et al. ^20^ states that capecitabine hyperpigmentation can be found in the palmar-plantar skin and oral mucosa, being rarer in the latter, even in patients with no history of HFS. Howhever other authors consider hyperpigmentation as an initial manifestation of the syndrome ^8^ ^,^ ^17^. Narasimham et al. ^21^ reports that the higher occurrence of HFS in black individuals suggests that this toxicity is more frequent in individuals from this population than in the white population. The authors also describe that in black individuals HFS grade 1 starts with progressive hyperpigmentation, and not acral erythema, involving the palms of the hands and soles of the feet, just as it was observed in the patient of this report. However, this suggestion is made based on clinical experience and not on controlled clinical trials.

The possibility that oral mucosa pigmentation be a distinct entity or that might be other manifestation of HFS has been the subject of discussion in the literature, because of the few reports describing oral mucosa pigmentation associated with HFS ^3^ ^,^ ^4^ ^,^ ^8^ ^,^ ^22^. Caprez et al. ^4^, rise the hypothesis that palmar-plantar hyperpigmentation from HFS and the oral mucosa hyperpigmentation by capecitabine administration are distinct entities, precisely due to the atypical pattern that these lesions present. In some situations, individuals have hyperpigmentation without a previous diagnosis of HFS ^20^. Other authors argue that oral stains occurs as an early manifestation of HFS, especially in the primary stages of the disease, thus constituting an important clinical predictor of HFS ^8^ ^,^ ^17^ ^,^ ^22^, as the case of aforementioned patient, whose oral lesions appeared before the grade 1 HFS clinical diagnosis. It should be noted that the patient reported no pigmentation of the oral mucosa prior to capecitabine therapy; moreover, oral mucosa pigmentation appeared months before the HFS diagnosis. This factor reinforces the theory of hyperpigmentation as sign that precedes HFS.

The diagnosis of drug-related hyperpigmentation is based on the relationship between the onset of signs and symptoms, the time of drug administration, and based on the exclusion of lesions that have a similar clinical appearance, but with a different etiology ^23^ ^,^ ^24^ ^,^ ^25^. In the present case, the macules were distributed on the buccal mucosa, gums, and palate with non-symmetrical and non-uniform dimensions. Although the patient in the present case is black and has a history of tobacco use, the relationship between the time of drug administration and the appearance of lesions, associated with the diagnosis of HFS, was essential to attribute the oral mucosa pigmentation to capecitabine therapy. Regarding the diagnosis of HFS, it is based on the clinical aspects; in this case, the patient presented dysesthesia, edema, dryness, pigmentation of the hands and nails, soles of the feet, and diarrhea. All these elements together favored the diagnosis of HFS.

The treatment of HES is basically symptomatic through the prescription of topical agents. The use of urea or lanolin-based protective ointments and the use of mild emollient creams is advisable. Other supportive measures such as the administration of topical and systemic antibiotics and the use of topical corticosteroids to decrease pain and inflammation may also be used. Despite this, the most appropriate management for the syndrome is the discontinuation of the drug. Complete healing of HFS is possible as long as localized ulceration does not occur ^14^ ^,^ ^25^.

Due to the clinical appearance of this lesion, the differential diagnosis with other pigmented lesions in the oral cavity is essential, and requires the involvement of the dentist in the multidisciplinary oncology team. Knowing the patient's medical history and understanding the oral repercussions of the various drugs used as antineoplastic therapy is the dentist's role, especially in cases of oral pigmentation where no or few therapeutic approaches are available^4^. In addition, it is also important to publish reports like this one, so that this knowledge can be more widely disseminated and, therefore, offer greater support to the proper dental management.

## Conclusion

Hyperpigmentation of the oral mucosa is a rare consequence of capecitabine antineoplastic therapy. Whether it is a part of an early manifestation of HFS or if it is an independent entity remains unclear. We report a case of hyperpigmentation of oral mucosa before the clinical diagnosis of HFS, which could be an early sign of this syndrome. New studies should be published in the literature in order to elucidate this process.

## References

[1] Roosendaal J, Jacobs BAW, Pluim D, Rosing H, de Vries N, van Werkhoven E (2020). Phase I pharmacological study of continuous chronomodulated
capecitabine treatment. Pharm Res.

[2] Lv H, Yan M, Zhang M, Niu L, Zeng H, Cui S (2014). Efficacy of capecitabine-based combination therapy and
single-agent capecitabine maintenance therapy in patients with metastatic
breast cancer. Chinese J Cancer Res.

[3] Vasudevan B (2010). An unusual case of capecitabine hyperpigmentation: Is
hyperpigmentation a part of hand-foot syndrome or a separate
entity. Indian J Pharmacol.

[4] Caprez J, Rahim U, Ansari A, Lodhi MU, Rahim M (2018). Hyperpigmentation with Capecitabine: Part of Hand-Foot Syndrome
or a Separate Entity?. Cureus.

[5] Lal HS (2014). Hand and foot syndrome secondary to capecitabine. Indian J Dermatol Venereol Leprol.

[6] Tavares-Bello R (2007). Capecitabine-induced hand-foot syndrome and cutaneous
hyperpigmentation in an elderly vitiligo patient [18]. J Eur Acad Dermatology Venereol.

[7] Agharbi FZ, Meziane M, Benhemmne H, Daoudi K, Elmesbahi O, Mikou O (2012). Hyperpigmentation à la capecitabine suivie de syndrome
mains-pieds: Une nouvelle observation. Ann Dermatol Venereol.

[8] Becker T (2015). Pigmented Lesions of Buccal and Labial Mucosa in a Patient
Treated by Capecitabine (Xeloda): A Case Report. Int J Dent Oral Heal.

[9] Lee SD, Kim HJ, Hwang SJ, Kim YJ, Nam SH, Kim BS (2007). Hand-foot syndrome with scleroderma-like change induced by the
oral capecitabine: A case report. Korean J Intern Med.

[10] Lou Y, Wang Q, Zheng J, Hu H, Liu L, Hong D (2016). Possible Pathways of Capecitabine-Induced Hand-Foot
Syndrome. Chem Res Toxicol.

[11] Yap YS, Kwok LL, Syn N, Chay WY, Chia JWK, Tham CK (2017). Predictors of hand-foot syndrome and pyridoxine for prevention of
capecitabine-induced hand-foot syndrome a randomized clinical
trial. JAMA Oncol.

[12] Nikolaou V, Syrigos K, Saif MW (2016). Incidence and implications of chemotherapy related hand-foot
syndrome. Expert Opin Drug Saf [Internet].

[13] Miller KK, Gorcey L, McLellan BN (2014). Chemotherapy-induced hand-foot syndrome and nail changes: A
review of clinical presentation, etiology, pathogenesis, and
management. J Am Acad Dermatol [Internet].

[14] Surjushe A, Vasani R, Medhekar S, Thakre M, Saple D (2009). Hand-foot syndrome due to capecitabine. Indian J Dermatol.

[15] Lassere Y, Hoff P (2004). Management of hand-foot syndrome in patients treated with
capecitabine (Xeloda®). Eur J Oncol Nurs.

[16] Abushullaih S, Saad ED, Munsell M, Hoff PM (2002). Incidence and severity of hand-foot syndrome in colorectal cancer
patients treated with capecitabine: A single-institution
experience. Cancer Invest.

[17] Sanghi S, Grewal RS, Vasudevan B, Nagure A (2013). Capecitabine induced hand-foot syndrome: Report of two
cases. Med J Armed Forces India.

[18] Da Cruz LA, Hoff PMG, Ferrari CLS, Riechelmann RSP (2012). Unilateral hand-foot syndrome: Does it take sides? Case report
and literature review. Clin Colorectal Cancer [Internet].

[9] Chen M, Chen J, Peng X, Xu Z, Shao J, Zhu Y (2017). The contribution of keratinocytes in capecitabine-stimulated
hand-foot-syndrome. Environ Toxicol Pharmacol [Internet].

[20] Tognetti L, Fimiani M, Rubegni P (2015). Benign dermoscopic parallel ridge pattern in plantar
hyperpigmentation due to capecitabine. Dermatol Pract Concept.

[21] Narasimhan P, Narasimhan S, Hitti IF, Rachita M (2004). Serious hand-and-foot syndrome in black patients treated with
capecitabine: Report of 3 cases and review of the literature. Cutis.

[22] Vickers MM, Easaw JC (2008). Palmar-plantar hyperpigmentation with capecitabine in adjuvant
colon cancer. J Gastrointest Cancer.

[23] Costa J dos S, Silva GM, Kameo SY, Amorim BF, Ramos MJO (2019). Síndrome Mão-Pé Induzida por Quimioterapia: Abordagem Clínica e
Epidemiológica de Pacientes com Câncer. Rev Bras Cancerol.

[24] Tosios KI, Kalogirou EM, Sklavounou A (2018). Drug-associated hyperpigmentation of the oral mucosa: report of
four cases. Oral Surg Oral Med Oral Pathol Oral Radiol [Internet].

[25] Treister NS, Magalnick D, Woo S Bin (2004). Oral mucosal pigmentation secondary to minocycline therapy:
Report of two cases and a review of the literature. Oral Surg Oral Med Oral Pathol Oral Radiol Endod.

